# The Nox2-ROS-Nlrp3 Inflammasome Signaling Stimulates in the Hematopoietic Stem/Progenitor Cells Lipogenesis to Facilitate Membrane Lipid Raft Formation

**DOI:** 10.1007/s12015-022-10481-2

**Published:** 2022-11-28

**Authors:** Ahmed Abdelbaset-Ismail, Andrzej K. Ciechanowicz, Kamila Bujko, Janina Ratajczak, Magdalena Kucia, Mariusz Z. Ratajczak

**Affiliations:** 1 Laboratory of Regenerative Medicine, Medical University of Warsaw, Warsaw, Poland; 2 Department of Surgery, Anesthesiology and Radiology, Faculty of Veterinary Medicine, Zagazig University, Zagazig, Egypt; 3 Stem Cell Institute at James Graham Brown Cancer Center, University of Louisville, 500 S. Floyd Street, Rm. 107, Louisville, KY 40202 USA; 4 Department of Regenerative Medicine, Center for Preclinical Studies and Technology, Medical University of Warsaw, Warsaw, Poland

**Keywords:** Nlrp3 inflammasome, Nox2, ROS, Membrane lipid rafts, Lipogenesis, Metabolism

## Abstract

Proliferation, metabolism, and migration of hematopoietic stem/progenitor cells (HSPCs) are coordinated by receptors expressed on outer cell membranes that are integrated into microdomains, known as membrane lipid rafts (MLRs). These structures float freely in the cell membrane bilayer and are enriched in cholesterol and sphingolipids for their functional integrity. Receptors, if expressed in MLRs, have prolonged occupancy on the cell surface and enhanced signaling power. Based on this, we have become interested in the regulation of synthesis of MLRs components in HSPCs. To address this, we tested the effect of selected factors that promote proliferation or migration and their potential involvement in the synthesis of MLRs components in HSPCs. Based on our previous research showing that HSPCs from Nox2-KO and Nlrp3-KO mice display a profound defect in MLRs formation, we focused on the role of Nox2-ROS-Nlrp3 inflammasome in regulating lipogenesis in HSPCs. We found that while at steady state conditions, Nox2-derived ROS is required for a proper expression of enzymes regulating lipogenesis, during inflammation, this effect is augmented by Nlrp3 inflammasome. Thus, our data sheds new light on the regulation of lipogenesis in HSPCs and the involvement of the Nox2-ROS-Nlrp3 inflammasome axis that differently regulates lipogenesis at steady state conditions and in response to inflammation, modulating MLRs-mediated responsiveness of these cells to external stimuli.

**Graphical Abstract:**

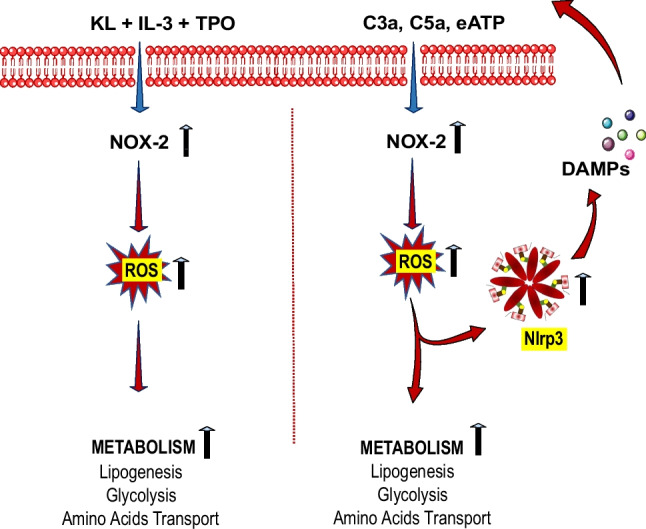

**Supplementary Information:**

The online version contains supplementary material available at 10.1007/s12015-022-10481-2.

## Introduction

Metabolism and migration of hematopoietic stem/progenitor cells (HSPCs) are coordinated by receptors regulating signaling expressed on cell outer membranes. These receptors are integrated into microdomains, known as membrane lipid rafts (MLRs) that float freely in the membrane bilayer and are enriched for cholesterol, sphingolipids, and ceramides for their functional integrity [1–3]. MLRs receptors include, e.g., the CXCR4 receptor for α-chemokine stromal-derived factor 1 (SDF-1), common ββ-subunit chain for IL-3, GM-CSF and IL-5 receptors, the c-kit receptor for stem cell factor (SCF), and the α1β4 integrin receptor (VLA-4) for vascular cell adhesion molecule-1 (VCAM-1), which all together regulate migration, proliferation, and adhesion of HSPCs [1, 3–5].

Based on this, we become interested in how stimulation of HSPCs by selected growth factors and cytokines as well mediators of inflammation affect lipogenesis in HSPCs to provide lipid components for MLRs. We envisioned that stimulation of lipogenesis in HSPCs and MLRs formation keeps these cells in an alert state to respond to external stimuli. Moreover, since we recently learned that Nlrp3-KO and Nox2-KO mice show a defect in MLRs assembly [5], we become interested in the potential role of Nox2-ROS-Nlrp3 inflammasome signaling in stimulating lipogenesis.

NADPH oxidase 2 (Nox2), as superoxide-generating enzyme, is a primary source of reactive oxygen species (ROS) that regulate intracellular redox state to modulate the proliferation and migration of HSPCs [6–8]. For many decades, ROS have been considered as cellular waste products within cells that can lead to oxidation events and permanent damage to DNA, lipids, and proteins. Recently, depending on the activation level, ROS are also recognized as “signaling molecules” having nuanced roles in regulating almost all aspects of cell function [9].

ROS follow a principle of hormesis where low activation of potentially harmful stimuli within so-called “hormetic zone” has a paradoxically beneficial effect on cell biology, in contrast to higher activation level outside the “hormetic zone” that leads to cellular aging, diseases, and death [4, 10, 11]. The most essential ROS is hydrogen peroxide (H_2_O_2_) that reversibly oxidizes redox-sensitive cysteine residues buried within transcription factors, enzymes, and structural proteins [9]. These ROS-mediated oxidative post-translational modifications control the transcription, expression, and biological activity of numerous enzymes as well as cellular localization of proteins and/or their interactions with binding partners. ROS-mediated “redox signaling” controls cell function by modifying the expression and activity of metabolic enzymes and transcription factors, including AKT kinases, CD39 and CD73 ectonucleotidases, NRF2, HIF-1α, FOXOs, HIF-1α, AP1, PTEN, SIRT1 [9, 12, 13]. What is important for our current report is that ROS also activates intracellular pattern recognition receptor Nlrp3 inflammasome [14] that has recently been proposed to regulate the proliferation of HSPCs in an interleukin-1β—dependent manner [4, 15, 16].

Since ROS are released in a Nox2-dependent manner from cells stimulated with various growth factors, cytokines, and pro-inflammatory mediators, to shed more light on the potential involvement of the Nox2-ROS-Nlrp3 inflammasome axis in lipid metabolism, we employed in our experiments HSPCs purified from normal and Nox2-KO animals and stimulated them with factors that do not activate directly Nlrp3 inflammasome (KL, IL-3, TPO) and factors that trigger this intracellular receptor during inflammation (C3a, C5a, and eATP) [17].

We provide novel evidence that Nox2-derived ROS are required for a proper expression of enzymes regulating metabolism in HSPCs at steady state conditions in Nlrp3 inflammasome-independent and during inflammation in a Nlrp3-dependent manner. This data establishes a novel link between innate immunity and cell metabolic pathways that supply lipid components for proper assembly of MLRs involved in migration, mobilization, homing, and engraftment of HSPCs.

## Materials and Methods

### Animals

Pathogen-free 6–8 weeks old C57BL/6 J mice (WT) and Nox2-KO were purchased at least 2 weeks before experiments from Jackson Laboratory, Maine, USA. Animal studies were approved by the University of Louisville (Louisville, KY, USA) and the Medical University of Warsaw (Warsaw, Poland).

### Isolation of SKL Cells

SKL cells were purified from the bone marrow (BM)-derived total nucleated cells (TNCs) of C57BL/6 wild-type and Nox2-knock out mice. In brief, BM was flushed from the femurs, and the population of TNCs was retrieved after lysis of red blood cells (RBCs) using Lyse buffer (BD Pharmingen, San Jose, CA, USA). Afterward, the harvested cells underwent staining with the following antibodies: lineage marker-specific antibodies: PE–anti-TCRγδ, clone GL3; PE–anti-CD11b, clone M1/70; PE–anti-TCR β-chain, clone H57–597; PE–anti-CD45R/B220, clone RA3-6B2; PE–anti-TER-119/erythroid cells, clone TER-119; PE–anti-Ly-6G and -Ly-6C (Gr1), clone RB6-8C5; PE–Cy5–anti-Ly-6A/E (Sca-1), clone D7; fluorescein isothiocyanate (FITC)–anti-CD117 (c-Kit), clone 2B8 in RPMI-1640 medium supplemented with 2% fetal bovine serum (FBS). All these antibodies were purchased from BD Biosciences. After 30 min of staining, the cells were washed, resuspended, and sorted using a Moflo XDP cell sorter (Beckman Coulter, Indianapolis, IN, USA) as populations of SKL (Sca-1^+^c-Kit^+^ Lin^−^) cells [18].

### Quantitative Real-Time PCR (RT-qPCR)

Cells were exposed to the hematopoietic growth factors and cytokines cocktail cytokines cocktail kit ligand (KL, Stem Cell Tech), recombinant murine interleukin 3 (mIL-3, PeproTech Rocky Hill, NY), and thrombopoietin (TPO, Stem Cell Tech) in serum-free medium for 1 h at 37 °C (RPMI-1640 / 0.5% BSA) at doses KL (1 ng/ml), mIL-3 (1 ng/ml), and thrombopoietin (TPO, 5 ng/ml). In the other experiments, cells were stimulated in vitro with NLRP3 inflammasome stimulators [C3a (1 μg/ml), C5a (1 μg/ml), or eATP (10 μM)] after pretreatment with or without MCC950, a NLRP3 inflammasome inhibitor, (10 μmol/l) in the assay medium. All control sets were only received a vehicle in the assay medium. Total RNA was extracted and purified from cells using the RNeasy Mini kit (Qiagen Inc., Valencia, CA, USA) after treatment with DNase I (Qiagen Inc.). Purified RNA was then reverse transcribed into cDNA with MultiScribe Reverse Transcriptase, oligo(dT), and a random hexamer primer mix (all from Applied Biosystems Life Technologies, CA, USA). Quantitative evaluation of the target gene was then performed by using an ABI Prism 7500 sequence detection system (Applied Biosystems Life Technologies) with Power SYBR Green PCR Master Mix reagent and specific primers (*mSREBP2: sense, 5′-gcgttctggagaccatgga-3′, antisense, 5′-acaaagttgctctgaaaacaaatca-3′; mGK *[19]*: sense, 5′-ccctgagtggcttacagttc-3′, antisense, 5′-acggatgtggagtgttgaagc-3′; mGLUT2 *[19]*: sense, 5′-cctcaagaggtaataatatccc-3′, antisense, 5′-ccatcaagagggctccagtc-3′; mHMGCR *[19]*: sense, 5′-cttgtggaatgccttgtgattg-3′, antisense, 5′- agccgaagcagcacatgat-3′; mHMGCS: sense, 5′-gccgtgaactgggtcgaa-3′, antisense, 5′-gcatatatagcaatgtctcctgcaa-3′; mPFKFB3 *[20]*: sense, 5′-ctatcccacgggagagtcc-3′, antisense, 5′-* tggcgctctaattccatga*-3′; mASMASE *[21]*: sense, 5′-tgggactcctttggatggg-3′, antisense, 5′- cggcgctatggcactgaat-3′; mG6PD *[22]*: sense, 5′-ccggaaactggctgtgcgct-3′, antisense, 5′-ccaggtcacccgatgcaccc-3′ and mSLC7A5/LAT1 *[23]*: sense, 5′-atatcacgctgctcaacggtg-3′, antisense, 5′-ctccagcatgtaggcgtagtc-3′).* The PCR cycling conditions were 95 °C (15 s), 40 cycles at 95 °C (15 s), and 60 °C (1 min). According to melting point analysis, only one PCR product was amplified under these conditions. The relative quantity of a target gene, normalized to the beta 2-microglobulin gene as the endogenous control (*B2M*: sense, 5′-catacgcctgcagagttaagca-3′, antisense, 5′-gatcacatgtctcgatcccagtag-3′) and relative to a calibrator, was expressed as 2^–ΔΔCt^ (fold difference).

### Isolation of Lipid Rafts

Isolation of fractions enriched with lipid rafts was performed using Caveolae/Rafts Isolation Kit (Catalog Number CS0750; Sigma-Aldrich, MO, USA). In brief, pre-cooled BM-mononuclear cells (5 × 10^7^ cells)—non-stimulated or stimulated with a cocktail of KL + IL-3 + TPO (as described above)—were centrifuged at 450* g* for 5 min at 4 °C, and then lysed in a Lysis Buffer (Catalog Number L7667) containing 1% Triton X-100 (Catalog Number X100) and protease inhibitors cocktail (Catalog Number P8340) for 1 h on ice. The density gradients were made of 5 layers (35%, 30%, 25%, 20%, and 0%) of OptiPrep density gradient medium (Catalog Number D1556) with lysis buffer containing 1% and cell lysate. 35% OptiPrep containing the cell lysate was placed at the bottom of the pre-cooled ultracentrifuge tube. Samples were then centrifuged at 200,000 g for 4 h at 4 °C using SW 55 Ti rotor (Beckman Coulter, Inc., CA, USA). Fractions were gently removed from the top of the gradients and stored at –20 °C. Western blot analysis was performed using standard techniques with rabbit anti-CXCR4 Polyclonal Antibody (Catalog Number PA5-19,856, Invitrogen) and rabbit Anti-Lyn Monoclonal Antibody (clone R.677.10, Catalog Number MA5-14,924, Invitrogen).

#### Glow Assay to Measure Activation of Nlrp3 Inflammasome

To measure the activity of caspase-1 in cells, Caspase-Glo® 1 Inflammasome Assay (Promega, USA) was employed, and analyses were performed according to the manufacturer’s protocols. Samples of control and cells exposed to C3a, C5a, KL + IL-3 + TPO, eATP were collected, and 10 × 10^5^ of BM MNCs were plated in 96 wells plate. Caspase-Glo® 1 Reagent or Caspase-Glo® 1 YVAD-CHO Reagent were added (100 μl/well), and luminescence was measured using a GloMax 9301 Multi Detection System after 90 min [24]. The doses and sources of factors employed for stimulation are described above.

#### Ultra-High Resolution Metabolomic Studies

The normalized number of FACS isolated SKL cells (9,000 cells) were lysed with 100 µl of ice cold (-20 °C) methanol (LC–MS hyper grade, Merck). The samples were incubated in -20 °C for 2 h during which the samples were thoroughly vortexed every 20 min. After this time, the samples were centrifuged (30 min, 18,000 × g, -10 °C,) and 80 µl of the supernatant was lyophilized with the use of a concentrator (Eppendorf). The lyophilizate was dissolved immediately before injection in a mixture of ddH_2_O and methanol (50:50 ratio) with addition of 0.1% formic acid. Untargeted metabolomics was analyzed using ultra-high-resolution Fourier-Transform Ion Cyclotron Resonance Mass Spectrometry with an electrospray ion source (ESI-FT-ICR-MS, SolariX 2xR 7 T, Bruker) in direct injection. The injection was made at a flow rate of 300 µl/h in both positive and negative polarity (with 5 technical replications of each sample). The ions accumulation time was set to 0.03 s., with the dry gas flow set to 4.0 L/min and drying temperature 200 °C. The capillary in the ion source was set to 3500 V (negative polarity) and 4500 V (positive polarity). The collected mass spectra (64 accumulated scans per spectrum) were analyzed using the T-Rex 2D algorithm (MRMS single spectra) in the MetaboScape 5.0 software (Bruker) and the identified compounds were assigned to specific signaling pathways using MetaboAnalyst 5.0 and the KEGG database.

### Lipidome

A normalized number of SKL cells at steady state conditions or stimulated by the cocktail of KL + IL-3 + TPO at doses described above (9,000 cells) was spined down (500xg, 20 min, 23 °C), and then the dried cell pellet was lysed firstly with freeze–thaw cycle (in -20 °C) and then with 225 µl of methanol (LC–MS hypergrade, Merck). Lysed cells were mixed with 750 µl tert-Butyl methyl ether (MTBE) and incubated on a shaker for 1 h at room temperature and 600 rpm. Then 160 µl of ddH_2_O was added to the samples. The samples were centrifuged at 1,000xg, for 10 min at 23 °C, then 100 µl of the organic phase was lyophilized. Before direct injection, the dried samples were dissolved in 500 µl of 100% methanol with 0.1% addition of formic acid.

Lipidome was analyzed using ESI-FT-ICR-MS (ultra-high-resolution Fourier-Transform Ion Cyclotron Resonance Mass Spectrometry with an electrospray ion source, SolariX 2xR 7 T, Bruker). The injection was made in 5 technical replications of each sample at a flow rate of 300 µl/h in both positive and negative polarity. The ions accumulation time was set to 0.02 s., with a drying temperature of 200 °C and the dry gas flow set to 4.0 L/min. The capillary in the ion source was set to 3500 V (negative polarity) and 4500 V (positive polarity). The collected mass spectra were analyzed in the MetaboScape 5.0 software (Bruker) using the T-Rex 2D algorithm (MRMS single spectra), and the identified compounds were assigned to specific signaling pathways using LipidMaps and the KEGG database.

### Statistical Analysis

Statistical analysis was performed using GraphPad Prism 6.0 (GraphPad Software Inc). All data are provided as an average ± SD. Statistical data analysis was performed using ANOVA and Tukey’s test for comparison between multiple groups and two samples, respectively. In all calculations, *p* < 0.05 and *p* < 0.01 were considered significant.

## Results

### Stimulation of HSPCs with Hematopoietic Growth Factors Stimulates Lipogenesis to Provide MLRs Lipid Components

**Fig. 1:**
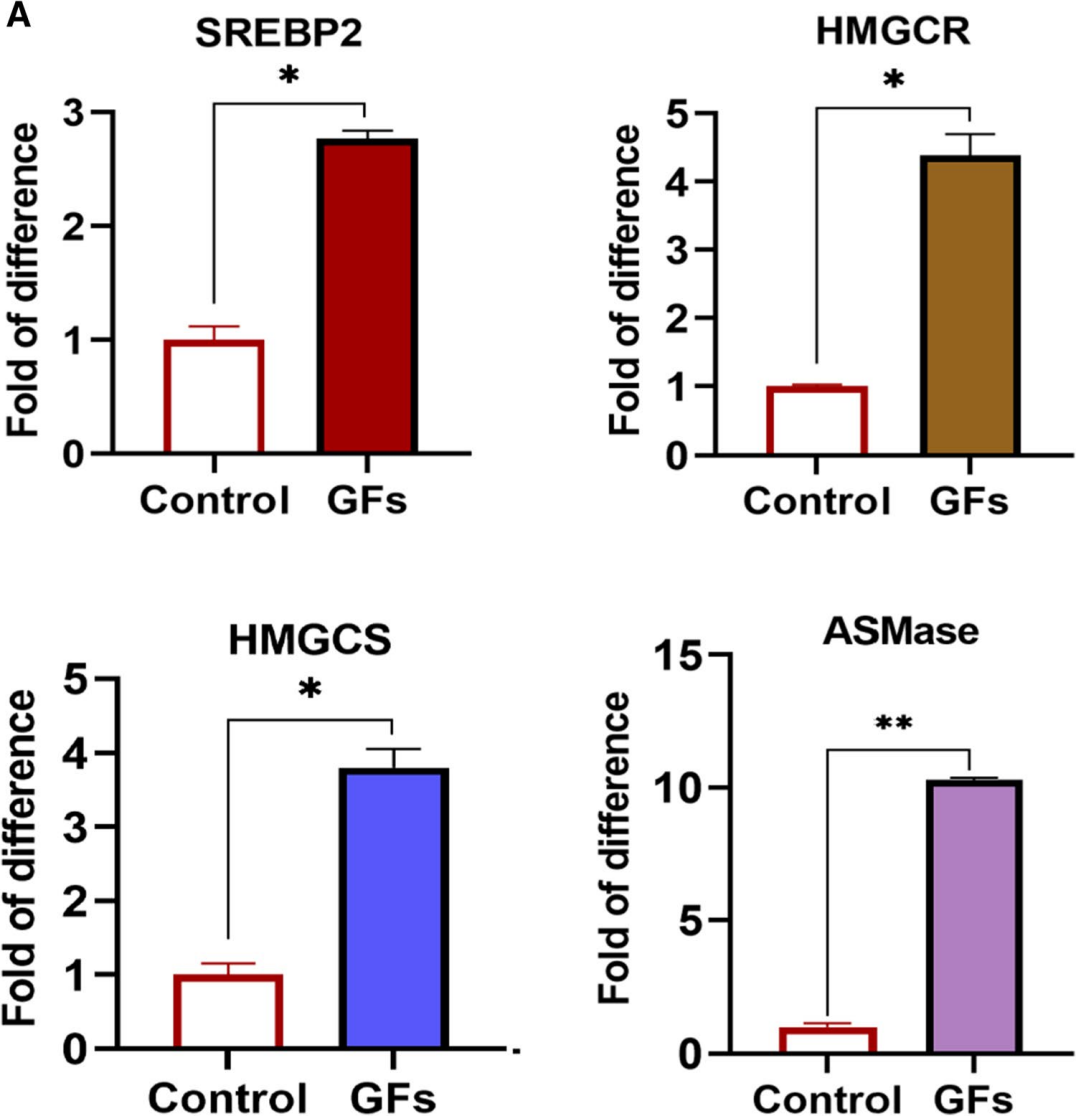

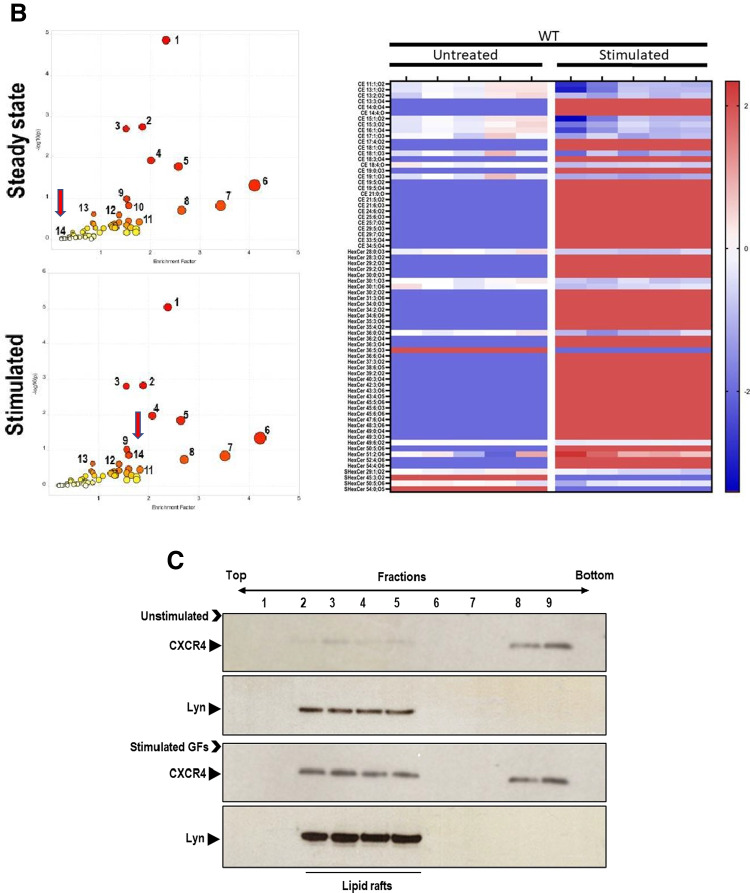
Stimulation of HSPCs with hematopoietic growth factors increases the expression of enzymes involved in cholesterol synthesis. Panel **A**. RT-qPCR analysis of the key enzymes involved in cholesterol synthesis [SREBP2, HMGCR, HMGCS, and ASMase]. mRNA samples were purified from HSPCs cultured with hematopoietic growth factors and cytokines cocktail [KL + IL-3 + TPO]: KL (1 ng/ml), mIL-3 (1 ng/ml), and thrombopoietin (TPO, 5 ng/ml) in a serum-free medium for 1 h at 37 °C. β2-microglobulin was used as an endogenous control. Non- stimulated HSPCs in serum-free medium were served as a control. *p < 0.05 and **p < 0.01 are considered statistically significant between cells exposed to hematopoietic growth factors and unstimulated cells. Samples containing only water instead of cDNA were used in each run as a negative control. Panel **B**. Untargeted metabolomic and lipidomic analysis. Panel B, left panel. Based on MetaboAnalyst 5.0 annotation of identified compounds to biological processes and signaling pathways, we analyzed differences between WT mice SKL cells in steady state condition (upper graph) and stimulated (bottom graph). Each number on the plot represents different biological process, i.e., 1.) Purine metabolism, 2.) Tryptophan metabolism, 3.) Metabolism of xenobiotics by cytochrome P450, 4.) Pentose and glucuronate interconversions, 5.) Folate biosynthesis, 6.) Thiamine metabolism, 7.) Phenylalanine, tyrosine, and tryptophan biosynthesis, 8.) Starch and sucrose metabolism, 9.) Drug metabolism – other enzymes, 10.) Amino sugar and nucleotide sugar metabolism, 11.) Galactose metabolism, 12.) Phenylalanine metabolism, 13.) Sulfur metabolism, 14.) Pentose phosphate pathway. Panel **B**, right panel. Heat map representing identified lipid compounds involved in lipid raft remodeling and structure. Abbreviations of the names of lipid compounds were used based on the LipidMaps classification. The heat map presents only statistically significant changes in lipid expression. Panel **C**. Western blot analysis of CXCR4 incorporation into lipid rafts within various fractions of cell membranes. The membranes enriched in lipid rafts are noted in fractions 2˗5. Murine mononuclear cells (MNCs) were cultured with [KL + IL-3 + TPO] in serum-free medium for 1 h at 37 °C. MNCs cultured only in serum-free medium served as control. CXCR4 expression was evaluated in the membrane fractions isolated from lysates of unstimulated (upper panel) and stimulated cells (lower panel) by western blot. Lyn, has been employed as a marker of lipid rafts

To address the effect of HSPCs stimulation on lipogenesis, we stimulated purified murine Sca-1^+^c-Kit^+^ Lin^−^ (SKL) cells with KL + IL-3 + TPO and evaluated the effect on mRNA levels for key enzymes involved in the synthesis of cholesterol. We noticed that all these factors stimulated the expression of mRNA for cholesterol synthesis key enzymes, including sterol regulatory element-binding protein 2 (SREBP2), 3-hydroxy-3-methyl-glutaryl-coenzyme A reductase (HMGCR), hydroxymethylglutaryl-CoA synthase (HMGCs), and acid sphingomyelinase (ASMAse) (Fig. 1A).

Interestingly at the same time, we also observed an increase in the expression of crucial enzymes involved in glycolysis – Glucokinase **(**GK), Glucose transporter 2 **(**GLUT2), Phosphofructokinase (PFKFB3), and Glucose-6-phosphate dehydrogenase **(**G6PD), as well as transmembrane amino acid transporter, large neutral amino acids transporter small subunit 1 (LAT1) (Supplementary Fig. 1).

### Metabolome Analysis Of Murine HSPCs Stimulated with KL + IL-3 + TPO Confirms Activation of Lipogenesis

We employed MetaboAnalyst 5.0 to identify compounds annotated to biological processes and signaling pathways and analyzed differences between SKL cells under steady state condition (Fig. 1B left upper panel) and stimulated with KL + IL-3 + TPO (Fig. 1B left lower panel). Each number on the plot represents different biological process, indicated by numeric numbers as described in the legend for this Figure. The most striking difference we observed in the case of the pentose phosphate pathway that provides NADPH required, e.g., for cholesterol and fatty acid synthesis—annotated as 14 and highlighted by red arrow. While cholesterol is incorporated into MLRs, fatty acids are utilized in sphingolipid synthesis [25].

Figure 1B right panel shows a heat map representing identified lipid compounds involved in MLRs formation and/or their rebuilding. We noticed upregulation of 19 isoforms changes in SKL cells stimulated with KL + IL-3 + TPO compared to unstimulated cells. Of note, among the identified cholesterol compounds, which appeared upregulated after stimulation, one of the cholesterol isoforms CE(14:0), also called 1-Myristoyl-cholesterol, is crucial important component of MLRs [26].

After SKL cell stimulation, we also observed the synthesis of cholesteryl nonadecanoic acid (CE(19:0)), which is the major transport and storage form of cholesterol in lipoprotein particles in most cell types. CE(19:0) is mediating the exchange in vivo of cholesteryl esters from circulating in blood HDL to MLRs [26, 27]. At the same time, cholesterol compounds that are not direct components of MLRs, but just a part of cell membrane lipoproteins were downregulated in stimulated cells. This included e.g., CE(11:1;O2), CE(13:1;O2) and CE(13:2;O2), which expression statistically significantly decreased by -1.33 fold change (p = 0.001), -1.31 fold change (p = 0.002) and -0.9 fold change (p = 0.02), respectively.

Moreover, we also observed a significant increase in the concentration of hexosyl ceramides belonging to sphingolipids that are crucial for MLRs assembly. These hexosyl ceramides serve as key precursors for the biosynthesis of dihexosyl derivates and more complex glycosphingolipids such as globosides and gangliosides enriched in MLRs. Upon stimulation of SKL cells with cocktail of KL + IL-3 + TPO, 31 hexosyl ceramides were identified as compared to non-stimulated SKL cells (Fig. 1B right panel**)**. Of note, the concentration of N-(hexadecanoyl)-1-β-glucosyl-4E,6E-tetradecasphingadienine (HexCer (30:2;O2), which interacts with TLR4 receptors embedded in MLRs [28] and N-(heneicosanoyl)-1β-glucosyl-4E,14Z-sphingadienine HexCer 39:2;O2) increased in stimulated cells [29]. Moreover, increased concentration of these compounds with the ability to spontaneously transfer lipids between cell membrane phospholipid bilayer, indicates a significant remodeling of the MLRs in stimulated SKL cells.

Figure 1C shows Western blot analysis of MLRs formation in BMMNC stimulated by KL + IL-3 + TPO and demonstrates CXCR4 receptor association with cell membrane fractions 2–5, that are enriched for MLRs.

### Activation of Nlrp3 Inflammasome in Stimulated Murine SKL Cells

**Fig. 2:**
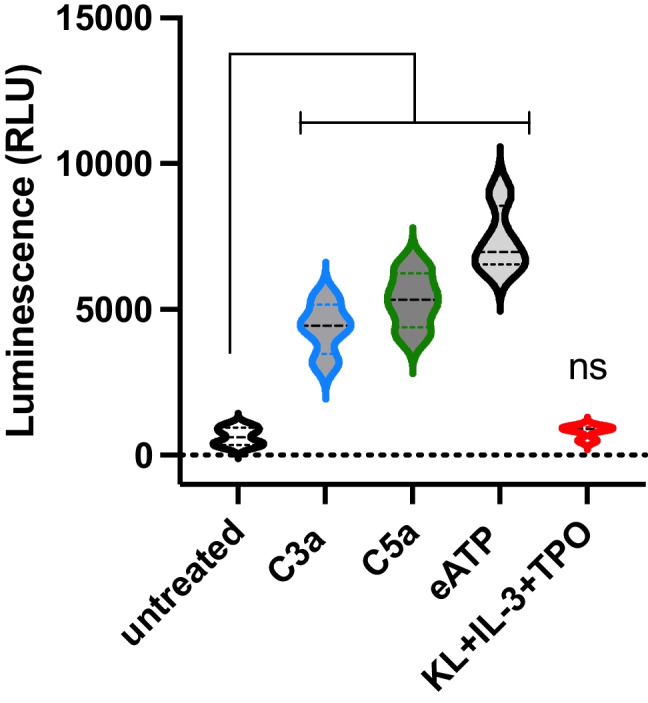
Activation of Nlrp3 inflammasome in stimulated murine SKL cells. Nlrp3 inflammasome activity was assessed by employing caspase-1 Caspase-Glo® 1 Inflammasome Assay (Promega, USA) in murine SKL cells exposed to C3a, C5a, eATP, or a cocktail of KL + IL-3 + TPO at doses described in Material and Methods section. Luminescence was measured using a GloMax 9301 Multi Detection System. Untreated SKL cells served as a control

HSPCs from Nlrp3-KO mice as mentioned have a profound defect in MLRs formation [5]. Therefore, to better assess the potential role of intracellular pattern recognition receptor Nlrp3 inflammasome in observed phenomena, we first stimulated murine SKL cells with active cleavage fragments of ComC—C3a and C5a and extracellular signaling nucleotide—adenosine triphosphate (eATP), as well as a cocktail of KL + IL-3 + TPO. In these stimulated cells, we measured activation of the Nlrp3 inflammasome by employing a glow assay (Fig. 2). We noticed that all the pro-inflammatory factors employed (C3a, C5a, and eATP), in contrast to KL, IL-3, and TPO, stimulated Nlrp3 inflammasome. Interestingly, to our surprise, we noticed an increase in intracellular expression of ROS (data not shown) that are known activators of this intracellular pattern recognition receptor, in both groups of stimulated cells. This data shows the requirement for ROS activation but simultaneously different utilization of ROS in activation of Nlrp3 inflammasome, pending which stimulating factors have been employed.

### The Expression of mRNA for Enzymes Involved in Cholesterol Synthesis, Glycolysis, and Amino Acid Transport is Nlrp3-Inflammasome Dependent in HSPCs Stimulated by C3a, C5a, and eATP

**Fig. 3:**
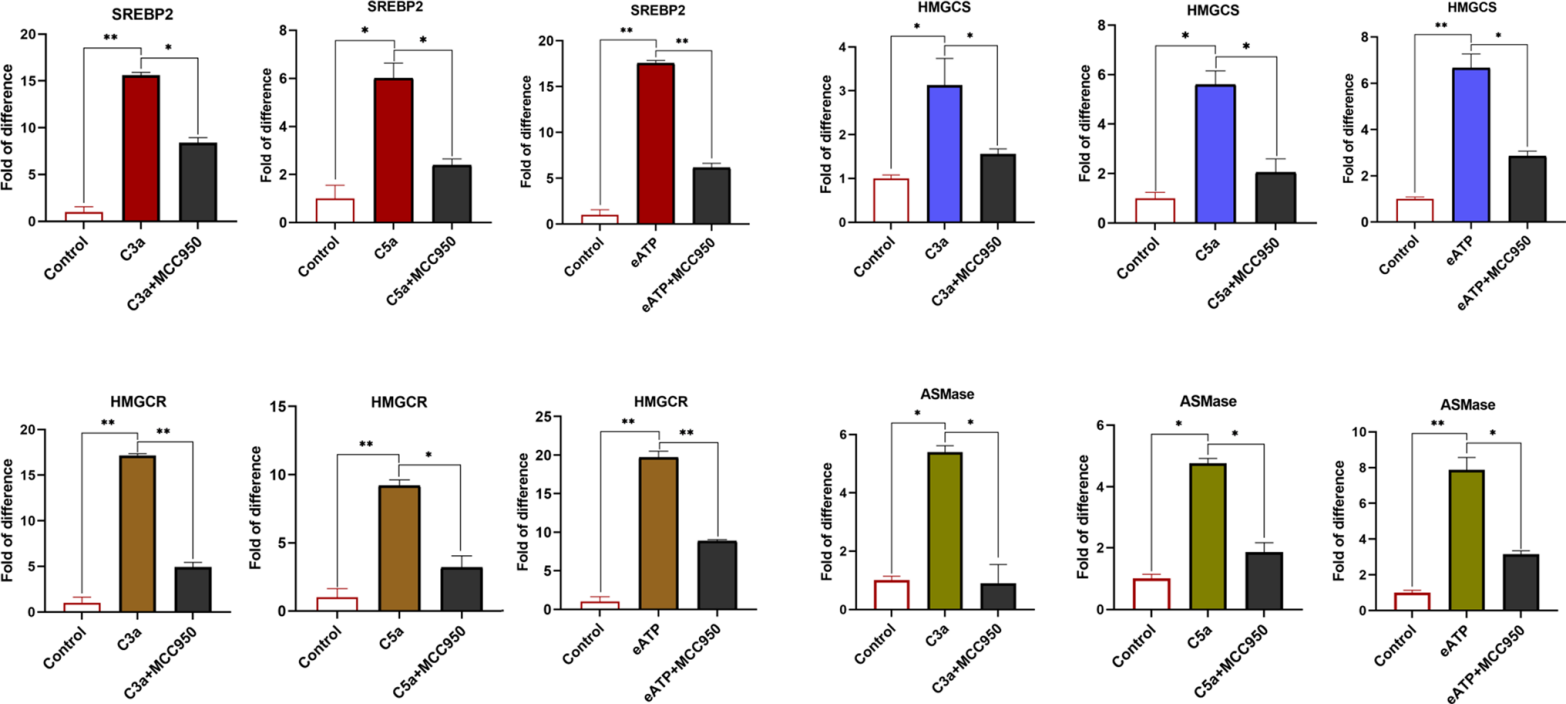
The expression of mRNAs for enzymes involved in cholesterol synthesis is Nlrp3-inflammasome dependent in HSPCs stimulated by C3a, C5a, and eATP. Expression analyses of the key enzymes involved in cholesterol synthesis [SREBP2, HMGCs, HMGCR, and ASMAse] were evaluated by qRT-PCR in mRNA samples purified from HSPCs cultured with NLRP3 inflammasome stimulators, C3a (1 μg/ml), C5a (1 μg/ml), or eATP (10 μM) in a serum-free medium for 1 h at 37 °C. Cells were preincubated for 1 h with or without MCC950 (10 μmol/l). For each experiment, β2-microglobulin was used as an endogenous control. Samples containing only water instead of cDNA were also used per each run as a negative control (data not shown). Untreated HSPCs cultured in only a serum-free medium were used as controls. Values of *p < 0.05 and **p < 0.01 are considered significant

Based on the Nlrp3 inflammasome activation study (Fig. 2), we asked if the increase in expression of enzymes involved in lipogenesis, glycolysis, and amino acid transportation is truly dependent on the activation of the Nlrp3 inflammasome. To address this question, we stimulated murine SKL cells with C3a, C5a, and eATP which are known activators of Nlrp3 inflammasome [17] (Fig. 3). We noticed that all these factors enhanced the expression of mRNA for key enzymes involved in cholesterol synthesis, including SREBP2, HMGCs, HMGCR, and ASMAse, and this effect was ameliorated if cells were exposed to Nlrp3 inflammasome specific inhibitor MCC950 as shown for C3a (Fig. 3A), C5a (Fig. 3B) and eATP (Fig. 3C). The similar effect we observed for enzymes involved in glycolysis GK, GLUT2, G6PD, and PFKFB3 (Supplementary Fig. 2) and amino acid transport LAT1 (Supplementary Fig. 3).

This data shows Nlrp3 inflammasome independent (Figs. 1 and 2) and Nlrp3 inflammasome dependent (Figs. 1 and 3) mechanism enhancing cell metabolism in HSPCs.

### The Effect of KL + TPO + IL-3 on the Expression of mRNA for Metabolic Enzymes in HSPCs is ROS-Dependent

**Fig. 4:**
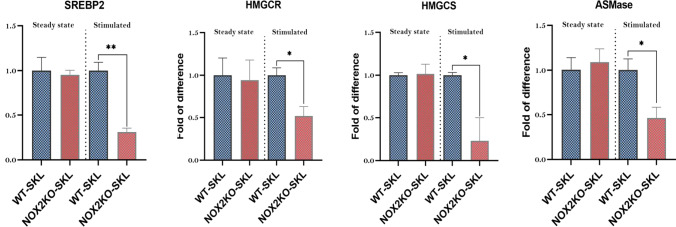
The effect of KL + TPO + IL-3 on the expression of mRNA for the key enzymes involved in cholesterol synthesis in HSPCs is ROS-dependent. RT-qPCR analysis of the key enzymes involved in cholesterol synthesis [SREBP2, HMGCs, HMGCR, and ASMAse] in mRNA samples extracted from HSPCs that were sorted by a Moflo XDP FACS cell sorter from bone marrows of both wild-type (WT) and Nox2-KO mice. The expression of these enzymes were initially assessed in a steady-state condition in SKL cells before giving any treatment (steady state pannels). This data in WT SKL cells are shown as to be 1.0 and changes in expression in cells from Nox2-KO mice are shown as relatively changes in expression to WT mice data. Next, HSPCs were exposed to hematopoietic growth factors and cytokines cocktail [KL (1 ng/ml) + mIL-3 (1 ng/ml) + thrombopoietin (TPO, 5 ng/ml)] in serum-free medium for 1 h at 37 °C. Per each run, β2-microglobulin was used as an endogenous control *p < 0.05 and **p < 0.01 are considered statistically significant

HSPCs from Nox2-KO mice as mentioned have a profound defect in MLRs assembly (ASH 2022 submitted). To shed more light on the role of the Nox2-ROS-Nlrp3 inflammasome axis in regulating the expression of enzymes involved in lipogenesis, we performed enzyme expression studies in HSPCs purified from normal wild-type and Nox2-KO mice that were non-stimulated or stimulated with KL + TPO + IL-3. As it is shown in Fig. 4, at steady state conditions in non-stimulated cells, we did not observe differences in the expression of enzymes involved in cholesterol synthesis SREBP2, HMGCs, HMGCR, and ASMAse. However, this changed upon stimulation with KL + TPO + IL-3 as we noticed a decrease in mRNA expression for all these enzymes in HSPCs purified from Nox2-KO mice indicating that ROS plays here an important role.

The similar effect we observed for enzymes involved in glycolysis GK, GLUT2, G6PD, and PFKFB3 as well as amino acid transport LAT1 (Supplementary Fig. 4).

## Discussion

**Fig. 5:**
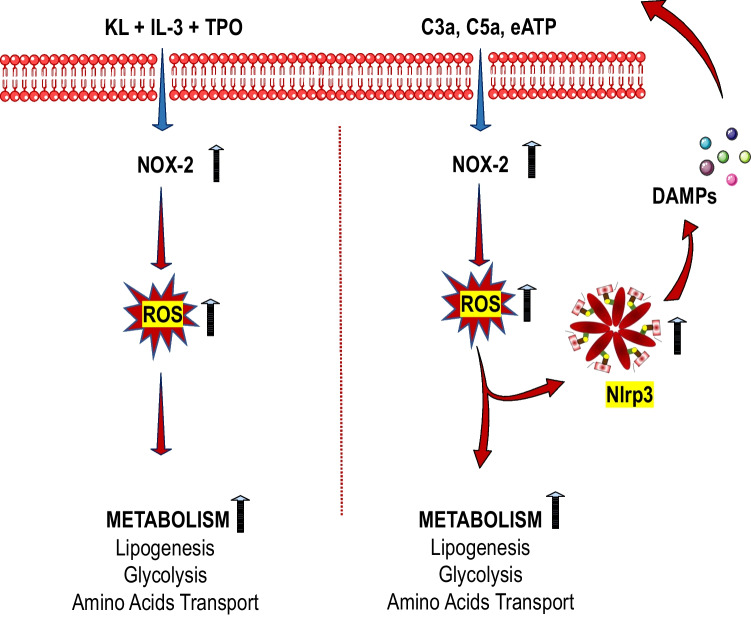
Differences in metabolical response of murine HSPCs stimulated by a mixture of hematopoietic growth factors and cytokines (KL + IL-3 + TPO) versus stimulation by pro-inflammatory mediators (C3a, C5a or eATP). A cocktail of KL + IL-3 + TPO activates the Nox2-ROS response to stimulate metabolism by upregulating enzymes involved in lipogenesis, glycolysis, and amino acid transport (left panel). In contrast, after stimulation by C3a, C5a or eATP is additionally activated Nlrp3 inflammasome that by releasing from cells DAMPs additionally potentiate Nox2-ROS stimulatory effect on metabolism after activation of corresponding DAMPs receptors

The seminal observation of this report is that upon activation of HSPCs, synthesis of MLRs lipid components increases. This keeps cells in an “alert state” to respond to external stimuli that regulate cell proliferation, differentiation, and trafficking [1]. Our data sheds new light on the regulation of lipogenesis in HSPCs and indicates the involvement of the Nox2-ROS-Nlrp3 inflammasome axis that differently regulates the metabolism and synthesis of lipids at steady state conditions and during inflammation. We report that while Nox2-derived ROS is required at steady state conditions for a proper expression of enzymes regulating lipogenesis for MLRs formation, and this occurs in Nlrp3 inflammasome-independent manner, during inflammation Nox2-ROS axis engages Nlrp3 inflammasome (Fig. 5). Based on this, we provide novel evidence on the regulation of lipogenesis in HSPCs by the Nox2-ROS-Nlrp3 inflammasome axis in steady-state conditions and in response to stressors.

First, we focused on the expression of mRNA for genes involved in cholesterol and sphingolipid synthesis, including SREBP2, which is a ubiquitously expressed transcription factor that controls cholesterol homeostasis by stimulating the transcription of sterol-regulated genes [30]. We also evaluated the expression of mRNA for HMGCR, that is the rate controlling enzyme of the mevalonate pathway that produces cholesterol and other isoprenoids [31–33], as well as the expression of mRNA for HMGCS, an enzyme that catalyzes the reaction in which acetyl-CoA condenses with acetoacetyl-CoA to form 3-hydroxy-3-methylglutaryl-CoA (HMG-CoA) [34, 35]. This reaction comprises the second step in the mevalonate dependent isoprenoid biosynthesis pathway, where HMG-CoA is an intermediate in both cholesterol synthesis and ketogenesis [36]. Finally, we studied mRNA expression for ASMase – an enzyme responsible for catalyzing the breakdown of sphingomyelin to ceramide and phosphorylcholine [37]. All these enzymes were upregulated in HSPCs stimulated with “cocktail of factors” that expand HSPCs such as KL + IL-3 + TPO, and by mainly pro-inflammatory factors including C3a, C5a, and eATP.

The expression of mRNA for enzymes involved in cholesterol and phospholipid synthesis was subsequently supported by our metabolome data showing an increase in pentose phosphate pathway. The pentose phosphate pathway is a metabolic pathway that generates NADPH required for cholesterol and fatty acid synthesis, as well as ribose 5-phosphate used in the synthesis of nucleotides and nucleic acids [38]. One of the uses of NADPH in the cell is in killing microbial infections and to prevent intracellular oxidative stress by converting reactive H_2_O_2_ into H_2_O by glutathione peroxidase [39].

MLRs related receptors include, e.g., the CXCR4 receptor, common β-subunit chain for IL-3, GM-CSF and IL-5 receptors, c-kit receptor), and the α1β4 integrin receptor (VLA-4), which all together regulate migration and proliferation of HSPCs [1, 40]. Our previous work demonstrated that MLRs are enriched on HSPCs after exposure to some mediators associated with inflammation, such as, e.g., C3a [41]. An important role in MLRs formation also played eATP mediated purinergic signaling activating Nlrp3 inflammasome [5]. We noticed that blockage of eATP releasing Pannex-1 channel or lack of Nlrp3 inflammasome has significant negative impact on MLRs formation [42]. However, the molecular explanation for these phenomena was not clear at that time. Herein, we provide evidence that HSPCs exposed to C3a, C5a, or eATP—known mediators of inflammatory response activate in cells synthesis of cholesterol and sphingolipids that are important components of MLRs. This response to pro-inflammatory mediators depends on Nlrp3 inflammasome activity as blockage of this pattern recognition receptor by specific inhibitor MCC950 negatively affected this effect in HSPCs.

A recently published paper demonstrated that Nlrp3 inflammasome stimulates hematopoiesis both in embryonic zebra fish HSPCs and promotes hematopoietic specification in cultures of induced pluripotent stem cells [43–45]. In the contrary, inhibition of Nlrp3 inflammasome inhibited HSPCs production [43]. This stimulatory role of the Nlrp3 inflammasome could be explained by the release of its product that is activated interleukin-1 beta (IL-1β) that may stimulate lipogenesis [43] as described in J774 monocyte-macrophage cell line [46] or primary macrophages [47]. Interestingly in this last paper [46], Nlrp3 inflammasome modulated macrophage glycolysis by increasing in an IL-1β-dependent manner the intracellular level of phosphofructokinase (PFKFB3). In this current paper, we confirmed that expression of this critical regulatory enzyme involved in glycolysis is truly Nlrp3 inflammasome dependent, as exposure of cells to MCC950 decreases the level of this enzyme in HSPCs. Nevertheless, one must to keep in mind that activation of Nlrp3 inflammasome in HSPCs releases in addition to active IL-1β also several danger associated molecular pattern molecules (DAMPs) or alarmin including eATP, HMGB-1, and S100A8/A9 [12, 17, 48] that by activating specific receptors may also affect cell metabolism. We postulate that this occurs in Nox2-ROS dependent manner.

As mentioned above, Nox2 as superoxide-generating enzyme supplies ROS to regulate the intracellular redox state and thus is involved in the proliferation and migration of hematopoietic stem/progenitor cells (HSPCs) [6, 8]. It is known that intracellular redox reactions involve a set of distinct molecular oxygen derivatives produced during aerobic metabolism and are intrinsically linked to intracellular energy metabolism. For many decades, ROS have been considered as cellular waste products within cells that can lead to oxidation events and permanent damage to DNA, lipids, and proteins. Recently, depending on the activation level, ROS are also recognized as “signaling molecules” having nuanced roles in regulating almost all aspects of cell function [6, 8, 9].

A low level of ROS activation within so-called “hormetic zone” has a beneficial effect on cell biology; however higher level of activation outside the “hormetic zone” leads to cellular aging, diseases, and death [9]. To explain these metabolic effects ROS reversibly oxidizes redox-sensitive cysteine residues buried within transcription factors, enzymes, and structural proteins [9]. These oxidative post-translational modifications control the transcription and expression and/or biological activity of numerous enzymes as well as intracellular localization of proteins and/or their interactions with binding partners.

ROS is also a known potent activator of Nlrp3 inflammasome [17]. This creates a positive feedback mechanism between Nox2-generated ROS and Nlrp3 inflammasome released DAMPs, which activate DAMP receptors and thus generate more cytosolic ROS. We postulate that the Nox2-ROS-Nlrp3 inflammasome axis plays an important role in stimulating cell metabolism both at steady state conditions as well as in response to inflammation (Fig. 5).

Cell metabolism is activated in response to growth factors and cytokines. It is why we noticed the activation of several enzymes activated in HSPCs after stimulation by KL + TPO + IL-3. Nevertheless, despite the increase in cytosolic ROS, the Nlrp3 inflammasome was not activated in these cells. It is why we postulate that Nox2-derived ROS regulates metabolism at steady state conditions in Nlrp3 inflammasome-independent and during inflammation in a Nlrp3-dependent manner (Fig. 5). Based on this, further research is needed to identify at the molecular level how ROS may interfere with expression at mRNA or activity enzymes investigated in this report. This could be the result of i) modification of transcription factors, ii) affecting the process of transcription and translation of mRNA into proteins, and iii) finally changing the protein structure of enzymes in an allosteric way [9].

To support this, it has been reported that exposure of HepG2 cells to ROS significantly increased mRNA expression for genes related to cholesterol synthesis and glucose uptake [19]. Moreover, ROS stimulated the proliferation of acute myeloid leukemia by upregulating glycolytic regulator phosphofructokinase (PFKFB3) [49]. To support this further, redox positively regulates by cysteine oxidation expression of, e.g., two crucial transcription factors NRF2 and HIF-1,α that promote the expression of several enzymes involved in glycolysis, pentose phosphate pathway, nucleotide biosynthesis, fatty acid synthesis, and glutathione synthesis and utilization [9]. This data is in agreement with our results that demonstrate the effect of Nox2-ROS signaling on the expression of enzymes involved in lipogenesis and glycolysis. Nevertheless, further studies are needed to shed more light on how various growth factors, cytokines, and DAMPs regulate these processes.

In conclusion, we provide evidence that cell metabolism in steady-state conditions as well as in response to inflammation is modulated in HSPCs by the Nox2-ROS-Nlrp3 inflammasome axis, and stimulation of lipogenesis modulates assembly of MLRs.

## Supplementary Information

Below is the link to the electronic supplementary material.

Supplementary file1 (PPTX 398 KB) Stimulation of HSPCs with hematopoietic growth factors increases the expression of enzymes involved in glycolysis and amino acid uptake. RT-qPCR analysis of the mRNA expression for key enzymes involved in glycolysis [GK, GLUT2, PFKFB3 and G6PD], and protein synthesis [SLC7A5/LAT1]. mRNA samples were purified from HSPCs cultured with hematopoietic growth factors and cytokines cocktail [KL+IL-3+TPO]: KL (1 ng/ml), mIL-3 (1 ng/ml) and thrombopoietin (TPO, 5 ng/ml) in a serum-free medium for 1 hour at 37 °C. β2-microglobulin was used as an endogenous control. Samples containing only water instead of cDNA were used in each run as a negative control. HSPCs without stimulation (only serum-free medium) served as a control. *p<0.05 and **p<0.01 are considered statistically significant between cells exposed to hematopoietic growth factors and unstimulated cells

Supplementary file2 (PPTX 1396 KB) The expression of mRNA for enzymes involved in glycolysis is Nlrp3-inflammasome dependent in HSPCs stimulated by C3a, C5a, and eATP. RT-qPCR analysis of mRNA expression of enzymes involved in glycolysis [GK, GLUT2, G6PD, and PFKFB3] in mRNA samples extracted from HSPCs cultured with either C3a (1 μg/ml), C5a (1 μg/ml), or eATP (10 μM) for 1 hour after treatment with or without MCC950 (10 μmol/l). For each experiment, β2-microglobulin was used as an endogenous control. Samples containing only water instead of cDNA were also used per each run as a negative control. Untreated HSPCs cultured in only a serum-free medium were used as controls. Values of *p<0.05 and **p<0.01 are considered significant

Supplementary file3 (PPTX 365 KB) The expression of mRNA for a key enzyme involved in amino acid transport, SLC7A5/LAT1, is Nlrp3-inflammasome dependent in HSPCs stimulated by C3a, C5a and eATP. RT-qPCR analysis of the enzyme potentially involved in protein synthesis, SLC7A5/LAT1, in mRNA samples purified from HSPCs cultured with C3a (1 μg/ml), C5a (1 μg/ml), or eATP (10 μM) for 1 hour after treatment with or without MCC950 (10 μmol/l). β2-microglobulin was used as an endogenous control. Samples containing only water instead of cDNA were also used per each run as a negative control. Untreated HSPCs cultured in only a serum-free medium were used as controls. Values of *p<0.05 and **p<0.01 are considered significant

Supplementary file4 (PPTX 1079 KB) The effect of KL + TPO + IL-3 on the expression of mRNA for enzymes involved in glycolysis and aminoacid uptake in HSPCs is ROS-dependent. RT-qPCR analysis of mRNA expression of enzymes involved in glycolysis [GK, GLUT2, G6PD, and PFKFB3], and amino acid transport [SLC7A5/LAT1] in mRNA samples extracted from HSPCs that were sorted by a Moflo XDP from the bone marrow of both wild-type (WT) and Nox2-KO mice. Expression of these enzymes were assessed in a steady-state condition in non-stimulated SKL cells SKL cells exposed to hematopoietic growth factors and cytokines cocktail [KL+IL-3+TPO]: KL (1 ng/ml), mIL-3 (1 ng/ml) and (TPO) 5 ng/ml) in serum-free medium for 1 hour at 37 °C. This data in WT SKL cells are shown as to be 1.0 and changes in expression in cells from Nox2-KO mice are shown as relatively changes in expression to WT mice data. Per each run, β2-microglobulin was used as an endogenous control, and cDNA-free samples (only water instead) were employed as a negative control. Unstimulated HSPCs cultivated in only serum-free medium were used as a control. *p<0.05 and **p<0.01 are considered statistically significant

## Data Availability

Raw data are available from authors upon request.
