# Supplementary figures and images for: The Nox2-ROS-Nlrp3 Inflammasome Signaling Stimulates in the Hematopoietic Stem/Progenitor Cells Lipogenesis to Facilitate Membrane Lipid Raft Formation
Source: Stem Cell Rev Rep. 2022 Nov 28;19(1):92–103. doi: 10.1007/s12015-022-10481-2 (PMC9823029; doi:10.1007/s12015-022-10481-2)

## Slide 1
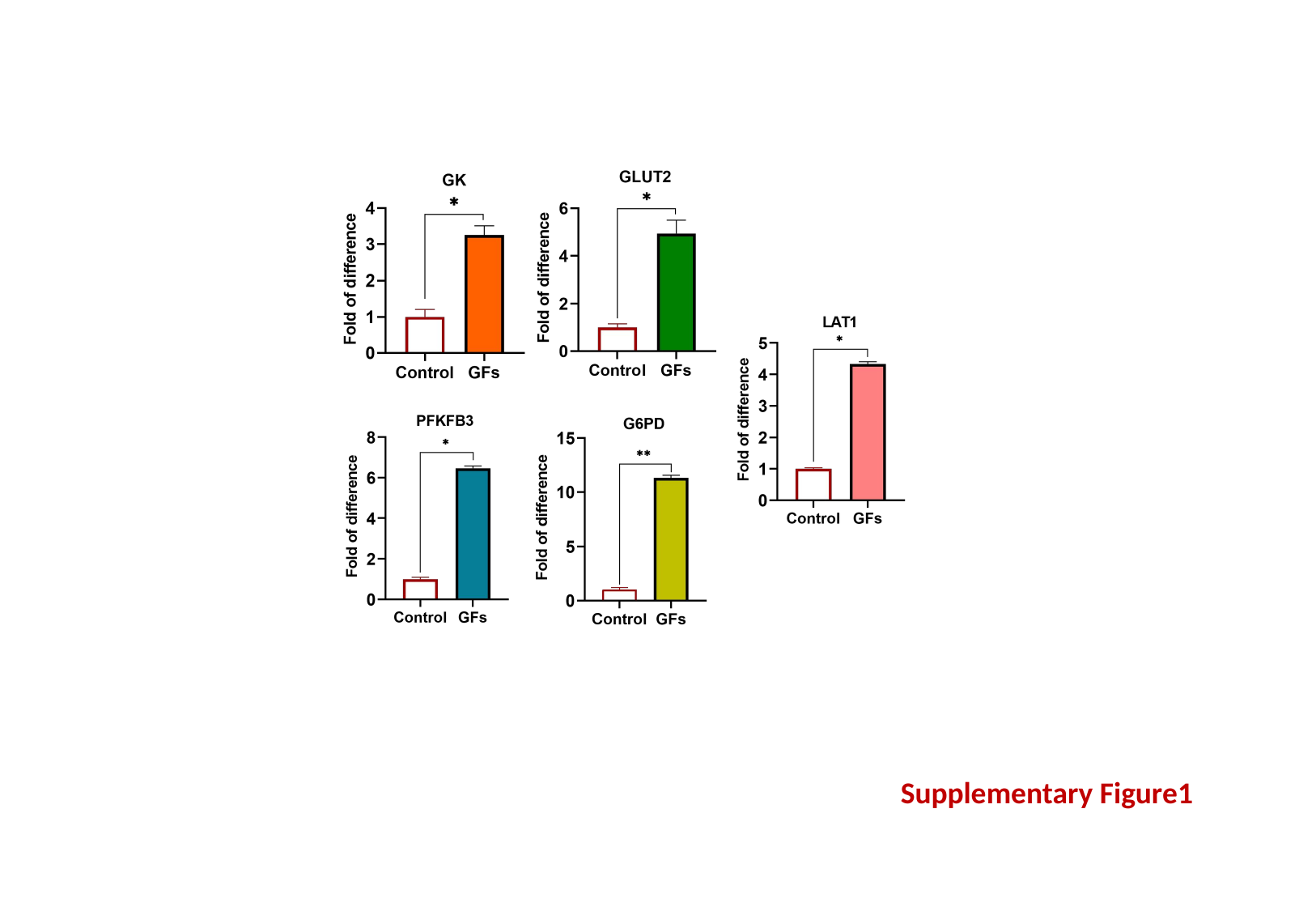

Supplementary Figure1

Supplement: Supplementary file 1 — Supplementary file1 (PPTX 398 KB) Stimulation of HSPCs with hematopoietic growth factors increases the expression of enzymes involved in glycolysis and amino acid uptake. RT-qPCR analysis of the mRNA expression for key enzymes involved in glycolysis [GK, GLUT2, PFKFB3 and G6PD], and protein synthesis [SLC7A5/LAT1]. mRNA samples were purified from HSPCs cultured with hematopoietic growth factors and cytokines cocktail [KL+IL-3+TPO]: KL (1 ng/ml), mIL-3 (1 ng/ml) and thrombopoietin (TPO, 5 ng/ml) in a serum-free medium for 1 hour at 37 °C. β2-microglobulin was used as an endogenous control. Samples containing only water instead of cDNA were used in each run as a negative control. HSPCs without stimulation (only serum-free medium) served as a control. *p<0.05 and **p<0.01 are considered statistically significant between cells exposed to hematopoietic growth factors and unstimulated cells [file 12015_2022_10481_MOESM1_ESM.pptx]

## Slide 1
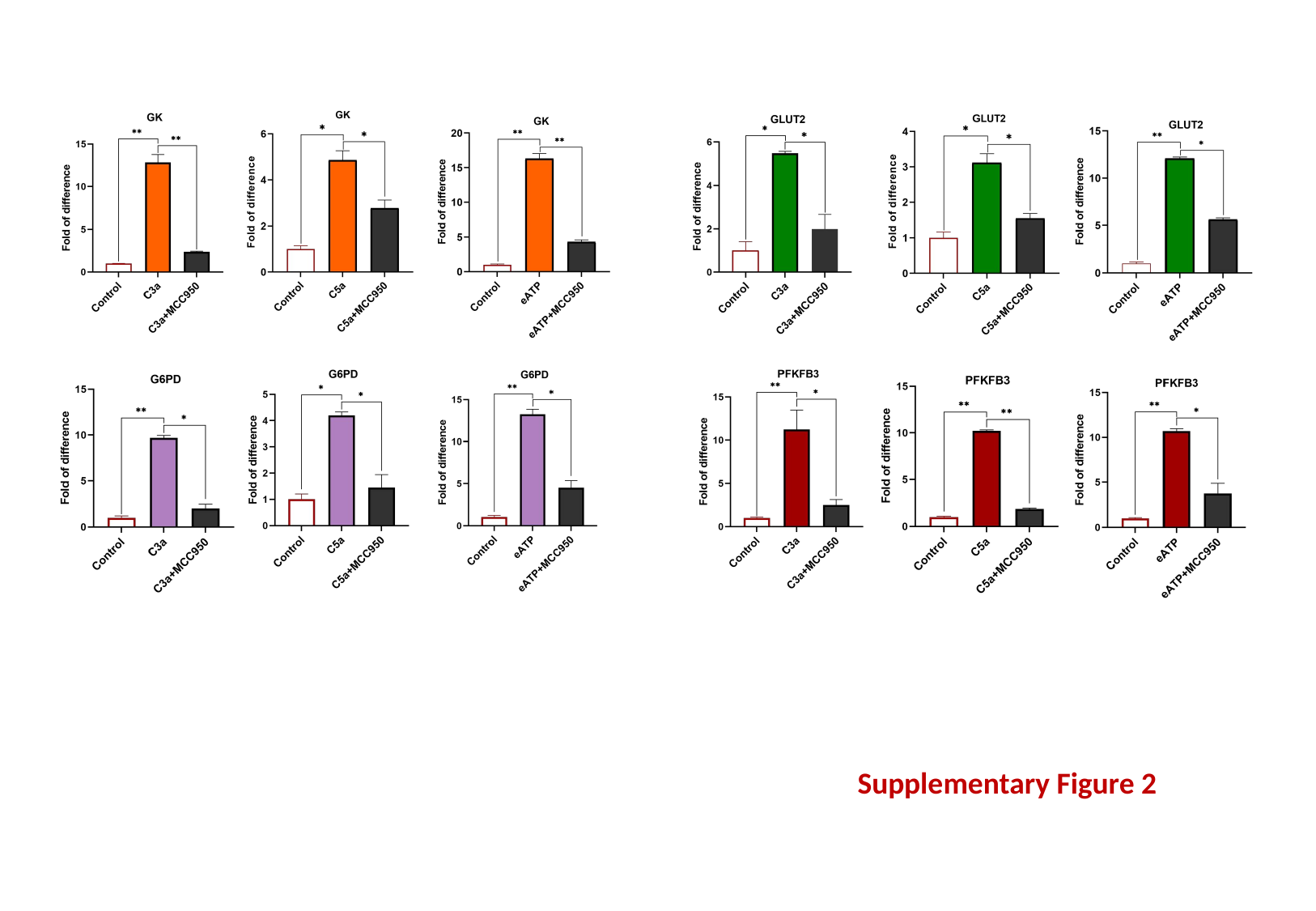

Supplementary Figure 2

Supplement: Supplementary file 2 — Supplementary file2 (PPTX 1396 KB) The expression of mRNA for enzymes involved in glycolysis is Nlrp3-inflammasome dependent in HSPCs stimulated by C3a, C5a, and eATP. RT-qPCR analysis of mRNA expression of enzymes involved in glycolysis [GK, GLUT2, G6PD, and PFKFB3] in mRNA samples extracted from HSPCs cultured with either C3a (1 μg/ml), C5a (1 μg/ml), or eATP (10 μM) for 1 hour after treatment with or without MCC950 (10 μmol/l). For each experiment, β2-microglobulin was used as an endogenous control. Samples containing only water instead of cDNA were also used per each run as a negative control. Untreated HSPCs cultured in only a serum-free medium were used as controls. Values of *p<0.05 and **p<0.01 are considered significant [file 12015_2022_10481_MOESM2_ESM.pptx]

## Slide 1
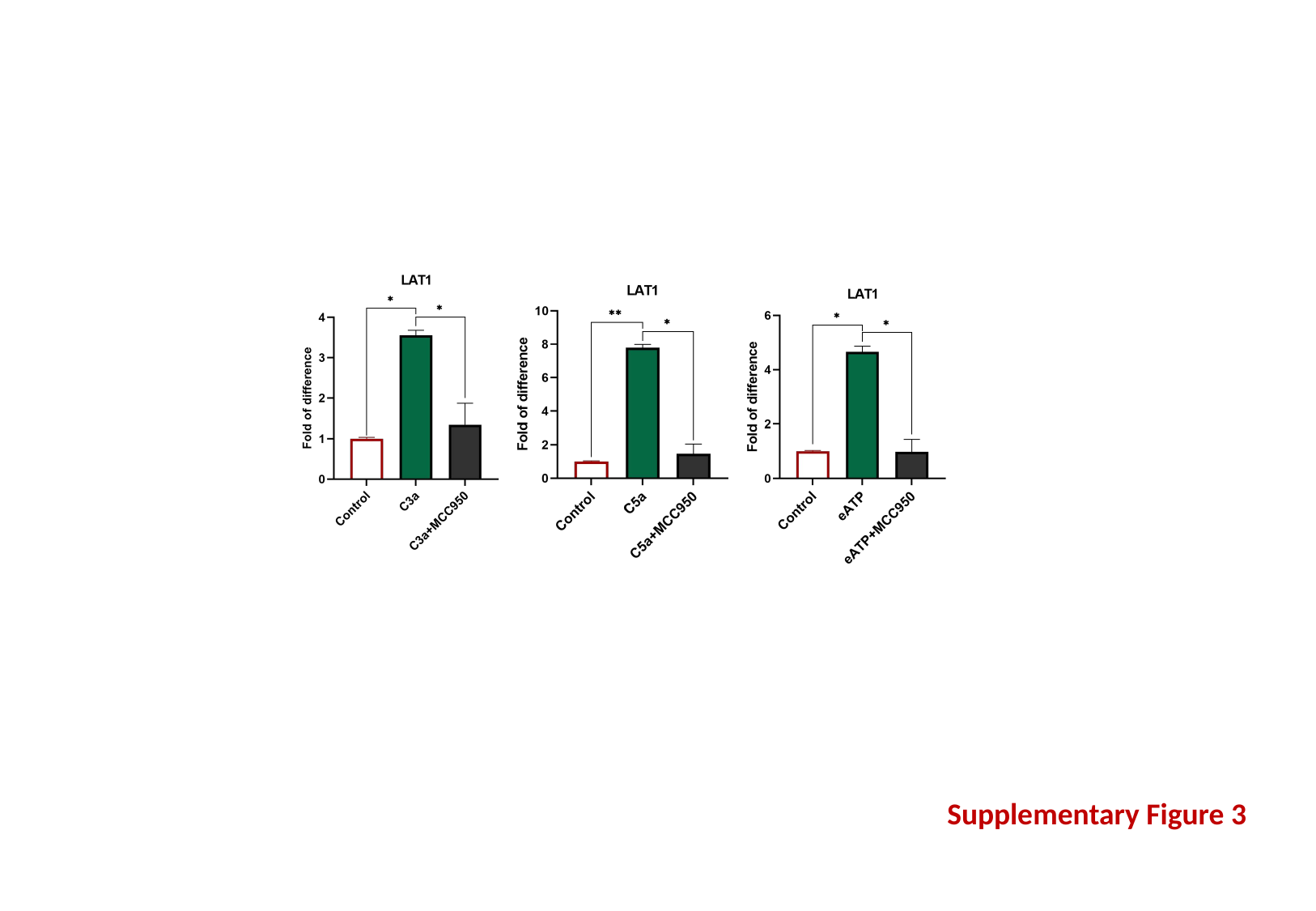

Supplementary Figure 3

Supplement: Supplementary file 3 — Supplementary file3 (PPTX 365 KB) The expression of mRNA for a key enzyme involved in amino acid transport, SLC7A5/LAT1, is Nlrp3-inflammasome dependent in HSPCs stimulated by C3a, C5a and eATP. RT-qPCR analysis of the enzyme potentially involved in protein synthesis, SLC7A5/LAT1, in mRNA samples purified from HSPCs cultured with C3a (1 μg/ml), C5a (1 μg/ml), or eATP (10 μM) for 1 hour after treatment with or without MCC950 (10 μmol/l). β2-microglobulin was used as an endogenous control. Samples containing only water instead of cDNA were also used per each run as a negative control. Untreated HSPCs cultured in only a serum-free medium were used as controls. Values of *p<0.05 and **p<0.01 are considered significant [file 12015_2022_10481_MOESM3_ESM.pptx]

## Slide 1
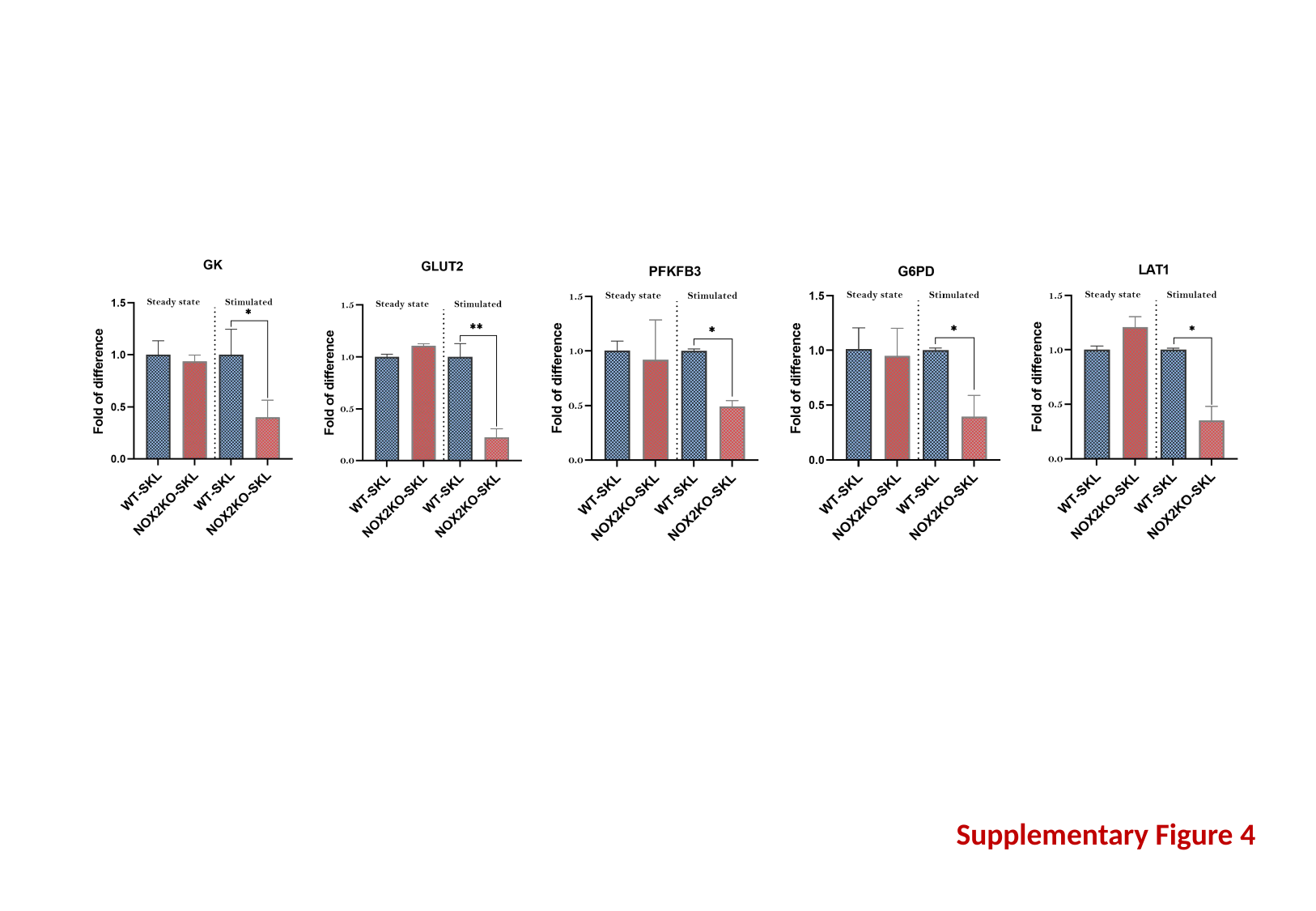

Supplementary Figure 4

Supplement: Supplementary file 4 — Supplementary file4 (PPTX 1079 KB) The effect of KL + TPO + IL-3 on the expression of mRNA for enzymes involved in glycolysis and aminoacid uptake in HSPCs is ROS-dependent. RT-qPCR analysis of mRNA expression of enzymes involved in glycolysis [GK, GLUT2, G6PD, and PFKFB3], and amino acid transport [SLC7A5/LAT1] in mRNA samples extracted from HSPCs that were sorted by a Moflo XDP from the bone marrow of both wild-type (WT) and Nox2-KO mice. Expression of these enzymes were assessed in a steady-state condition in non-stimulated SKL cells SKL cells exposed to hematopoietic growth factors and cytokines cocktail [KL+IL-3+TPO]: KL (1 ng/ml), mIL-3 (1 ng/ml) and (TPO) 5 ng/ml) in serum-free medium for 1 hour at 37 °C. This data in WT SKL cells are shown as to be 1.0 and changes in expression in cells from Nox2-KO mice are shown as relatively changes in expression to WT mice data. Per each run, β2-microglobulin was used as an endogenous control, and cDNA-free samples (only water instead) were employed as a negative control. Unstimulated HSPCs cultivated in only serum-free medium were used as a control. *p<0.05 and **p<0.01 are considered statistically significant [file 12015_2022_10481_MOESM4_ESM.pptx]
